# Bringing a genomic perspective to the safety of drug treatment in oncology

**DOI:** 10.12688/f1000research.10475.1

**Published:** 2017-03-29

**Authors:** Federico Innocenti

**Affiliations:** 1 Eshelman School of Pharmacy, Center for Pharmacogenomics and Individualized Therapy, Lineberger Comprehensive Cancer Center, University of North Carolina, Chapel Hill, NC, USA

**Keywords:** oncology, genetics, genomics, anti-cancer therapies, toxicity

## Abstract

This article describes the clinical relevance of toxicity of therapies administered to patients with cancer, putting the patient, rather than disease, at the center of the evaluation of safety of anti-cancer therapy. Hence, the implications of adverse events are described from the patient perspective, focusing on the impact of patient safety on quality of life and efficacy of treatment. Issues revolving around other types of safety, such as financial toxicity, are also discussed. The role played by genetics in the assessment of a patient’s risk of adverse events is also discussed, both in relation to the potential of genomic research and in the context of current tools of fruition in clinical care.

## Genomic perspective to the safety of drug treatment in oncology

By putting the patient, rather than the disease, at the center of personalized care, the concept of safety gains a much wider connotation. Hippocrates’s *primum non nocere* (“first, do no harm”) remains a fundamental guiding principle of any intervention in medical care. The suffering and discomfort experienced by patients because of symptoms related to the adverse events of drug treatment should never be discounted. The incidence of the symptoms, their severity, and the distress that they cause are often underestimated by physicians and other health professionals, compared with the symptoms reported by the patient ^1, 2^. Reasons for under-reporting of adverse reactions by clinicians (which for many symptoms has been estimated in more than 50% of patients) relate both to clinician factors (symptoms are considered unrelated to treatment or less attention is paid in reporting them) and to more complex factors related to patient-clinician interaction and communication, in addition to other reasons related to current grading systems ^3^.

Adverse events have a significant burden on the quality of life of the patient and family. They reduce confidence in the treatment and might demoralize the patient. We should not forget that, in this era of advanced technologies integrated into patient care, patients who receive anti-cancer therapies are still at risk of losing their lives as a result of the medication that should treat their cancers. A recent analysis of treatment-related mortalities of patients with cancer in clinical trials conducted in Europe has indicated that the rate of these catastrophic events is 0.7% (255 out of 34,734 patients) ^4^. This percentage is in line with the 0.5% of fatal toxicities observed in monotherapy phase I trials in oncology ^5^. Even with strict eligibility criteria for patient enrollment into trials, there is a lack of markers that can exclude high-risk patients from being treated. Clearly, the patient clinical characteristics are not sufficiently predictive for most experimental drugs, and genetic markers of life-threatening toxicities should be identified with urgency. Given that, in the context of clinical trials, patients are monitored more closely and intensively than in common practice, these so-called “toxic deaths” are still a reality. Patients are informed by the treating physician about the risk of treatment, and such risks include death. Patients might be willing to take any risk in light of a benefit from the treatment, but I consider toxic deaths ethically unacceptable, in particular when they are the consequence of the application of standard treatment regimens.

Severe adverse events often lead to permanent discontinuation of therapy. Mild to moderate toxicities, in addition to affecting quality of life, can reduce the intensity of the regimen. Most patients with cancer receive multi-drug regimens, and toxicity from one drug can halt the whole regimen until the functions of the affected organ have recovered. For drug regimens with curative intent—for example, acute lymphoblastic leukemia in children—preservation of dose intensity of the combination regimen increases the likelihood of long-term remission during the maintenance phase of treatment ^6^. When the goal of treatment is to maintain acceptable quality of life and prolong survivorship (or survival), patient discomfort (from gastrointestinal effects) or cosmetic changes (acne or skin rash), even when they are not severe, might reduce adherence to oral therapy, increasing the chance of recurrence of disease. This paradigm applies also to chemotherapy administered intravenously ^7, 8^. It could be foreseen that in different treatment scenarios—for example, in the two extremes of adjuvant versus palliative care—the risk/benefit pendulum would swing toward either benefit or risk, and this evaluation becomes context-dependent.

The risk of adverse events increases in the elderly, and risk assessment tools have been devised to predict such risk ^9^. It should be kept in mind that in this population, the risk in itself, without any tools to predict it, is often a deterrent to the administration of cancer therapies because of concerns about the capacity of elderly patients to endure treatment. Even in this patient setting, avoiding treatment in otherwise eligible patients is ethically questionable if the risk/benefit ratio favors prolongation of life or amelioration of the quality of life of a patient.

“Financial toxicity” adds another element to patient safety in oncology. The term refers to the financial burden of patients who experience out-of-pocket expenses for their treatments ^10^. I envision that, in the US, the expected changes in health care by the new administration ^11^, combined with the approval of expensive medications, will result in significant financial toxicity for patients. To defray out-of-pocket expenses, increased financial distress will negatively alter patient care.

Even for effective drugs like immunotherapies that are considered relatively safe, severe (albeit rare) adverse events can be recognized in the post-marketing phase of their development. Very recently, fatal, fulminant myocarditis of nivolumab and ipilimumab was reported in two patients with melanoma ^12^. In these patients, selective clonal T cell populations within the myocardium were identical to those detected in the tumor. This finding suggests a host reaction at the basis of the pathophysiology of the T cell clone recognition, the genetic underpinning of which remains to be established.

Genetic analyses of patient DNA have the potential of improving the safety of cancer drugs in several ways. If we consider lack of efficacy as the most clinically relevant adverse effect of cancer treatment, tailored therapies are already administered to patients selected on the basis of tumor DNA profiling, to improve the likelihood of response. In addition to the tumor DNA variation, the risk of developing severe adverse effects after standard doses of chemotherapy can be established by using the germline DNA variation of the host. Genotyping of patient germline DNA can be obtained before therapy, to assess whether such genetic predisposition exists ^13^. Most genetic biomarkers of drug safety have been identified after evidence has been achieved on various domains ^14– 16^. These domains include the elucidation of the pharmacological properties of the drug, knowledge of the main disposition pathways linking drug exposure to the occurrence of the adverse effects, obtaining functional validation of the genetic marker *in vitro*, establishing the analytical validity of the genotyping assay, and demonstration of clinical validity and utility ^17^.

When access to the genetic profile of the patient is possible, a better evidence-based decision on the type and intensity of treatment can be achieved for an individual patient. This has particular value when multiple regimens that are similarly effective are available, and both efficacy and safety can be maximized through the guide of genetic biomarkers. In fact, the number of oncology drugs approved by the US Food and Drug Administration is continually growing, leading to an even more compelling need of biomarkers to navigate an increasing number of therapeutic options. Access to genetic profiling is becoming more common for patients. Genetic profiling can be obtained at the level of germline DNA (heritable variations) and tumor DNA (somatic, non-heritable variations). Aside from the use of direct-to-consumer testing, patients with cancer might benefit from genetic screen of their tumor DNAs, which can be obtained through commercial products or as part of clinical trials of targeted therapies assigned on the basis of sequencing of the tumor’s DNA ^13^. These screens might include a matching germline DNA sample in addition to the tumor DNA sample. Traditionally, germline variation informs increased risk of toxicity, whereas somatic variation informs the driver gene to select the targeted therapy. Even when these genetic data are collected as part of a research protocol, it can be envisioned that if the results of the genetic screen are stored in the patient’s electronic medical record, use of the stored genetic changes (through validation of the molecular alteration by orthogonal assays) can be applied to the anti-cancer regimen to be administered. With the advent of liquid biopsies for the analysis of somatic mutations in cell-free DNA, such collection of potentially informative biomarkers is expected to increase even further ^18^. Stored genetic data can also inform the selection of future regimens to improve the safety of therapies to be given in the event of tumor recurrence or progression.

In the research space, comprehensive interrogations of the germline DNA of patients with cancer include genome-wide association studies and exome/genome sequencing ^19^. The characterization of hundreds of thousands of genetic changes in the genome allows the identification of new genes related to the pharmacology of the cancer drug or the pathophysiology of the adverse event or both. The investigation of these novel genes in experimental systems will guide the discovery of genetic changes likely to predispose the patient to increased risk of an adverse event. It will also lead to the development of supportive therapies that will be designed to modulate the new biological pathways identified by the genomic analysis.
